# Physical Performance and Quality of Life of Nursing-Home Residents with Mild and Moderate Dementia

**DOI:** 10.3390/ijerph10126672

**Published:** 2013-12-02

**Authors:** Elisabeth Wiken Telenius, Knut Engedal, Astrid Bergland

**Affiliations:** 1 Faculty of Health Sciences, Oslo and Akershus University College of Applied Sciences; Oslo 0130, Norway; E-Mail: astrid.bergland@hioa.no; 2 Oslo University Hospital, Ageing and Health: Norwegian Centre for Research, Education and Service Development, Oslo 0424, Norway; E-Mail: knut.engedal@aldringoghelse.no

**Keywords:** quality of life, dementia, nursing home, balance, mobility, muscle strength and activity of daily living

## Abstract

*Introduction*: The aims of this study were to describe the quality of life (QoL) of nursing-home residents with dementia and their balance, mobility, muscle strength and daily life activity, as well as to examine the associations between QoL and levels of balance, mobility, muscle strength and daily life activity. *Methods*: The study is cross sectional, and 170 nursing-home residents with dementia were included. *Tests:* “The quality of life in late-stage dementia scale” (QUALID), Berg Balance Scale, comfortable walking speed, maximum walking speed, 30-s sit-to-stand, Barthel Index, Clinical Dementia Rating Scale, the Clock Drawing Test and the Mini-Mental State Examination (MMSE) were used. *Results*: Our study showed that nursing-home residents with dementia are a heterogeneous group regarding registrations of QUALID and physical function measures. The scores on the QUALID ranged from 11 to 41 points. Higher scores on the 30-s sit-to-stand and Berg Balance Scale were associated with a better QUALID. For comfortable, as well as maximum, walking speed there was a trend towards better QUALID results for those participants with higher walking speed. *Conclusions*: Good muscle strength and balance were the most important physical performance variables significantly associated with a good QUALID score.

## 1. Introduction

Physical health and function are not the only desirable outcomes for nursing home residents with dementia. Because many dementia patients must live in a nursing home for an extended period of time, their quality of life (QoL) is equally important as their physical health [1]. According to Cooney and colleagues, the focus in the gerontology literature has been quality of care rather than QoL for these residents [2]. However, it is evident that the QoL of the elderly in nursing home is a determinant of their satisfaction with life [3,4] as well as a measure of their general health [5,6]. QoL has become a major topic in dementia research to improve the care of these patients [7,8,9,10,11,12,13,14,15].

QoL is defined by the World Health Organization as: “individuals’ perceptions of their position in life in the context of the culture and value system in which they live and in relationship to their goals, expectations and standards” [16]. Quality of life is a multifactorial concept. According to WHO, QOL is “affected by physical health, psychological state, level of independence, social relationship, personal beliefs and relationship to salient features of their environment” [16]. Zahn [17] states that QOL is a difficult construct to define and measure because cultural, ethical, religious, and other personal values infuence the perceptions of meaning of QOL. Another difficulty is the lack of consensus regarding QoL in persons with dementia [18]. Lawton’s model [9] includes both subjective and objective factors based on the following four components: behavioural competence, objective environment, psychological well-being and perceived QoL. There is a consensus that the subjective component is important to the concept of QoL; however, there is uncertainty about the ability of a patient with dementia to communicate reliably about their QoL. Compared to people with dementia who live at home, similar patients in institutions have greater difficulties reporting their perceptions about their own QoL because of more advanced cognitive impairments, such as severe memory and communication problems [19]. They also have less insight into their own situation [20].

Studies have found that the QoL is diminished among elderly patients in nursing homes compared to elderly persons staying at home [21,22]. Suggested reasons behind this difference are limited privacy and isolation [23], chronic diseases, loss of control and lack of autonomy, which may lead to helplessness, depression and apathy [24]. Many observational studies on younger and older adults have reported the beneficial effects of physical exercise on the quality of life, mood, satisfaction and emotional wellbeing [25]. Active people feel better than their sedentary counterparts [26,27,28]. Individuals who have moved into a nursing home are at risk of becoming even more sedentary, and Dechamps and colleagues describe them as having “an extremely sedentary lifestyle” [29]. A study reported that up to 94% of nursing home residents spend their time sitting or lying down, in spite of the fact that some of them are capable of independent activities such as walking [30]. Studies have suggested that greater dependency due to physical and mental impairment among institutionalised older people is inversely related to health-related QoL [16,31,32].

It is important to identify modifiable factors underlying or associated with QoL. The importance of physical activity for the preservation of function in elderly people is well documented [33], and it has been shown that muscle strength, mobility, balance, ability to perform the Activities of Daily Living (ADL), and cognitive function [34,35,36,37,38,39,40] can be improved through exercise [41].

Functional status affects the QoL in old age [42]. Older people, who are physically active and spend less time sitting down, experience higher levels of QoL than those who are less active [43]. In both rehabilitation research and practice, there is widespread recognition that physical function is a key component in the evaluation of health and QoL in older people [44]. Physical-performance measurements are of special interest because they can identify a decline in physical function and because they are modifiable, they can provide a foundation for the implementation of preventive measures [45,46]. Because performance-based tests are more sensitive than self-reported measures of physical function, it may be better to use them to follow the progression of a condition and to prescribe intervention for each individual [47].

QoL has been examined in nursing homes [21,48,49], but many previous studies have excluded patients with dementia and have not examined the associations between QoL and performance-based observation of physical functioning. To date, no study appears to have reported on the relationship between QoL and the physical performance of nursing-home residents with dementia. Karinkanta and colleagues did a cross-sectional study on home-dwelling older women with dementia and reported that muscle strength was significantly associated with QoL [50]. Yümin and colleagues studied the impact of functional mobility and balance on health-related QoL among elderly people living at home and those living in nursing homes [48]. They found a significant relationship between QoL and the Berg Balance Scale (BBS) in the participants living at home but not in those living in nursing homes. Their findings concur with those of Karinkanta and colleagues [50] and Ozkan and colleagues [51], who investigated the relationship between risk factors for falling and QoL in nursing-home residents. They found an association between QOL and balance, strength and mobility. Dementia diagnosis was an exclusion criterion in the studies of Yümin and colleagues [49] and Ozkan and colleagues [51]. Thus, there is a need to explore this area further, including persons with dementia. 

In the absence of a cure for dementia, there is an increasing focus on improving the QoL as well as the performance level of persons with dementia, as this could be one of the most important outcomes of interventions for this group of patients. Yet, as far as we know, there is no published research on the association between QoL and physical functioning for nursing-home residents with dementia, as well as the association between various measurements of performance-based physical function. Therefore, the aim of the study was to describe the QoL of nursing-home residents with dementia and their physical performance levels of balance, mobility, muscle strength and activity of daily living as well as to examine the associations between QoL and physical performance levels of balance, mobility, muscle strength and activity of daily living. With support from the literature, we hypothesise that QoL is significantly associated with physical performance among residents with dementia in nursing homes.

## 2. Methods

### 2.1. Design

This study is cross sectional. The data were collected from a convenience sample based on baseline measurement in a randomised controlled trial that was carried out in 2012 and 2013 in Norway. Eighteen nursing homes in Oslo, as well as in the counties of Akershus and Buskerud participated.

### 2.2. The Participants

We included 170 nursing-home residents, and the inclusion criteria were the following: having dementia of a mild or moderate degree as defined by a score of 1 or 2 on the Clinical Dementia Rating Scale [52]; age above 55 years; able to stand up with help from another person; able to walk six meters with or without a walking aid; and able to give informed consent. The exclusion criteria were the following: patients with medically unstable disorders that were unable to take part in physical activities and patients with long-term psychotic or severe communication problems. The nursing staff found suitable participants, between six and 12 persons at each nursing home. A total of 182 persons agreed to participate, however eight changed their mind prior to first assessment and four were excluded because exclusion criteria were not met. This gives a drop-out rate of 6.6%.

### 2.3. Ethical and Legal Considerations

The study was approved by the Regional Committee for Medical Ethics in Norway. Written and verbal information about the study was given to the patients and their relatives by their primary care giver. The participants gave their consent to participate and were informed that they could refuse to participate at any stage in the study.

### 2.4. Measurements

Chronic and current physical disorders were recorded and organised into 10 categories, e.g., cardiovascular disorders, endocrine disorders, neoplasm, disorders in the eyes, ears, genitourinary organs, respiratory organs, digestive organs, musculoskeletal disorders, and others.

“The quality of life in late-stage dementia scale”, QUALID [53], a proxy-rated scale was used to measure the QoL. The informants were professional caregivers who knew the patient well and had spent at least 3 of the last 7 days with the patient. The scale consists of 11 questions regarding the patient’s behaviour, mood and well-being. It explores to which degree the participant enjoys touch/touching, eating and interaction with others, and how often he/she smiles, appear sad or in discomfort, irritable or emotionally calm. Each question has five possible answers from never/seldom to every day. Total possible score range from 11 to 55. A lower score indicates a higher quality of life. It assesses both positive states and behavioural engagement. Previous studies among nursing-home residents in Scandinavia have shown good inter-rater and internal reliability in the use of this scale [22]. It is validated for nursing home residents with dementia [54].

*Balance* was measured by the Berg Balance Scale (BBS). The BBS is a performance-based measure of balance consisting of 14 observable tasks frequently encountered in everyday life. Scoring is based on the patient’s ability to perform the 14 items or movements independently and meet certain time and distance requirements. The test is simple and easy to administer and is safe for the elderly to perform. The test assesses performance on a 5-level scale from 0 (cannot perform) to 4 (normal performance) on 14 different tasks involving functional balance control, including transfer, turning and stepping [55]. The total score ranges from 0 to 56. Scoring is based on the ability to perform items independently and to meet given time or distance requirements. The scale has been shown to possess very good intra-rater and inter-rater reliability when used with an elderly population [55,56,57]. Acceptable validity estimates have also been reported [58].

*Mobility* was measured by the six-meter walking test, which includes comfortable speed and maximum walking speed, with or without a walking aid. The time in seconds was recorded and calculated as meters per second [59].

*Muscle strength* was measured by the 30-s sit-to-stand test (30-s chair stands). The score equals the number of rises from the chair in 30 s with arms folded across the chest [60].

To measure the patients’ dependence/independence in the Activities of Daily Living (ADL), we employed the Barthel Index (BI), a widely used measure of the activities of daily living [61]. The BI consists of 10 activities focusing on the patient’s level of dependence on aid. The scores range from 0 (completely dependent) to 20 (independent) [62]. The total maximum score of 20 implies that the patient can eat, attend to personal hygiene, get dressed, go to the bathroom, walk at least 50 m and use stairs. Professional care-givers filled out the BI-questionnaire based on their observations of the participants.

Rating Scale (CDR), the Clock Drawing Test (CDT) and the Mini-Mental State Examination (MMSE). CDR is a six-point scale used to characterise six domains of cognitive and functional performance applicable to Alzheimer’s disease and related dementias [52,63,64]. Two Norwegian studies have shown that CDR staging is a valid substitute for a dementia assessment among nursing-home patients to rate dementia and dementia severity [63,64]. The MMSE was used to assess global cognition and consists of items concerning orientation, word registration and recall, attention, naming, reading, writing, following commands and figure copying. It can be scored between zero and 30, where a higher score indicates better performance [65]. In addition, we used the Norwegian revised version of the Mini-Mental State Examination (MMSE-NR) [66]. The test has shown satisfactory reliability [67,68]. CDT was used to evaluate visuo-constructive abilities [21]. The patients are presented with a piece of paper with a pre-drawn circle and are asked to insert the numbers so the circle looks like the face of a clock, and then to draw the hands of the clock to read “10 past 11”. We used the 6-point scoring system as described by Shulman [69], with a range of 0–5 points, where 5 points is best.

### 2.5. Demographic Factors

The demographic factors taken into account are participant age, gender and previous and present medical history, and the length of stay in a nursing home (from date of admission).

### 2.6. Procedure

The physical function testing was performed by research physiotherapists. Before the study was initiated, testers from the different research sites took part in a training program on testing procedures to ensure high inter-rater test reliability. The cognitive tests were administered by nursing-home employees who had previous experience in using these tests.

### 2.7. Statistics

All statistical analyses were conducted with the SPSS Statistical Software version 20.0 for Windows. Data are presented with proportions and percentages for categorical values and means with standard deviation (SD) for interval data. The chi square test was used to compare categorical data, and Student’s t-test was applied for interval data. Correlation analyses (Pearson’s R) were conducted to examine the associations between the physical function variables in order to discover multicollinearity. To evaluate the crude associations between the demographic data, physical performance and health variables measured at baseline, and with the QUALID outcome as the dependent variable, we used unadjusted linear regression analyses. Furthermore, to adjust for possible confounders, we selected variables having the strongest association with the outcome (*p* < 0.05) from the unadjusted analyses and fitted multiple linear regression models, in addition to the variables of age and gender. Two different multiple regression models were fitted because of a high correlation (multicollinearity, see Table 2) between the two physical performance predictor variables (“30-s chair stand” was included in the first model and BBS in the second model). This measure was also taken to identify a model that explained the largest proportion of the variance in the QUALID. To compare the strength of the association between the various possible predictors and the main outcome (QUALID), we used the standardised betas from the regression models with their p-values. In all analyses, the level of statistical significance was set at *p* < 0.05, and all tests were two-tailed.

## 3. Results

Of the 170 residents with dementia, 125 (73.5%) were women with a mean age of 88.2 (SD = 6.0) years. Characteristics from the demographic data and concerning the health of the eligible participants are shown in Table 1.

The age of the participants ranged between 60 and 100 years; 25% were 82 years or older; and the youngest participants had the best results on BBS (r = −0.16, *p* = 0.04). The mean duration of stay in nursing home for the whole population was 2 years and 2 months (SD = 24.5). On average the men’s stays were 6 months longer. Almost one out of two suffered from cardiovascular disease and 20% had a psychiatric diagnosis. About one in three suffered from a neurological condition and cardiovascular accident was most common (n = 27).

The men were significantly younger than the women, had a lower frequency of diagnosed musculoskeletal disease and achieved better results on the “30-s chair stands”, as well as a higher maximum walking speed, compared with the women. Approximately 50% of the participants used a zimmer frame, and 10% used a wheelchair. The participants’ scores on the MMSE (n = 147) ranged from 2 to 28 points.

**Table 1:** Characteristics of demographic data, health and physical function variables for whole sample and according to gender.

	Whole Sample (n = 170)	Women (n = 125)	Men (n = 45)	*p*-Value *
Age in years mean (SD)	86.9 (7.36)	88.24 (6.0)	83.2 (9.4)	0.000
Use of walking aid, n (%)	118 (69.4)	90 (72.0)	28 (62.2)	0.22
Able to rise from chair independently, n (%)	158 (92.9)	116 (92.8)	42 (93.3)	0.81
Musculoskeletal diagnosis, n (%)	59 (34.7)	52 (41.6)	7 (15.5)	0.003
Neurological diagnosis, n (%)	31 (18.3)	20 (16.0)	11 (24.4)	0.22
Cardiovascular disorders, n (%)	79 (46.5)	58 (46.4)	21 (46.7)	0.22
Psychiatric diagnosis, n (%)	34 (20)	28 (22.4)	6 (13.3)	0.19
Mini-Mental State Examination score in points, mean (SD) *	15.6 (4.9)	15.5 (4.6)	15.8 (5.8)	0.59
Clock drawing test, mean (SD)	1.9 (1.6)	2.0 (1.6)	1.7 (1.5)	0.30
Duration of stay in nursing home (months), mean (SD)	25.7 (24.5)	23.9 (22.9)	30.9 (28.2)	0.12
Number of chronic disorders, mean (SD)	3.4 (1.9)	3.5 (2.0)	3.2 (1.7)	0.26
Number of drugs, mean (SD)	6.4 (3.4)	6.2 (3.3)	7.0 (3.5)	0.12
Berg Balance Scale in points, mean (SD)	34.7 (14.1)	34.1 (13.6)	34.1 (15.2)	0.43
30-s sit-to-stand (number of rises from chair), mean (SD)	6.1 (3.0)	5.8 (2.9)	6.8 (3.2)	0.05
Comfortable walking speed in m/s, mean (SD)	0.5 (0.2)	0.5 (0.2)	0.5 (0.2)	0.13
Maximum walking speed in m/s, mean (SD)	0.8 (0.3)	0.7 (0.3)	0.9 (0.4)	0.006
Barthel Index in points, mean (SD)	13.6 (3.7)	13.8 (3.6)	13.2 (4.0)	0.37
QUALID, mean (SD)	18.1 (5.8)	18.3 (5.8)	17.5 (5.6)	0.44

*p* = level of significance; * based on independent *t*-test between gender; SD = Standard deviation.

**Table 2:** Correlation between the different physical function measures and QUALID (The Pearsons Correlation Co-efficients).

	Berg Balance Scale	30-s sit-to-stand	Comfortable walking speed	Maximum walking speed	Barthel Index
	(n = 166)	(n = 167)	(n = 166)	(n = 166)	(n = 162)
30-s sit-to-stand	0.70				
(n = 167)	(*p* < 0.01)				
Comfortable walking speed	0.63	0.66			
(n = 166)	(*p* <0.01)	(*p* < 0.01)			
Maximum walking speed	0.66	0.67	0.77		
(n = 166)	(*p* <0.01)	(*p* < 0.01)	(*p* < 0.01)		
Barthel Index	0.66	0.59	0.53	0.55	
(n = 162)	(*p* <0.01)	(*p* < 0.01)	(*p* < 0.01)	(*p* < 0.01)	
QUALID	−0.2	−0.2	−0.14	−0.13	−0.078
(n = 168)	(*p* =0.01)	(*p* = 0.01)	(*p* = 0.08)	(*p* = 0.09)	(*p* = 0.33)

**Table 3:** Linear unadjusted and adjusted regression analyses * of the association between QUALID (dependent variable) and variables measuring demographic factors, health and physical performance.

Independent Variables	Unadjusted Analysis		Adjusted Analysis *		Adjusted Analysis **	
	Model 1		Model 2		Model 3	
	β	*p*	β	*p*	β	*p*
Age in years	0.08	0.30	0.06	0.49	0.03	0.69
Gender (men = 1, women = 0)	−0.06	0.44	−0.006	0.94	−0.05	0.54
Use of walking aid (yes = 1, no = 0)	0.04	0.66				
MMSE	−0.09	0.3				
Clock drawing test	−0.05	0.56				
Duration of stay in nursing home (months)	0.08	0.35				
Number of diagnoses	−0.02	0.85				
Number of drugs	0.06	0.48				
Musculoskeletal diagnosis	0.07	0.38				
Neurological diagnosis	−0.06	0.42				
Cardiovascular disorders	−0.02	0.77				
Psychiatric diagnosis	0.11	0.16				
Berg Balance Scale (points)	−0.20	0.01			−0.19	0.016
30-s sit-to-stand	−0.20	0.01	−0.20	0.01		
Comfortable walking speed	−0.14	0.08				
Maximum walking speed	−0.13	0.09				
Barthel Index	−0.08	0.33				

β = Standardized coefficient; * Not including Berg’s Balance Scale because of the level of its correlation (Pearson correlationcoefficient = 0.71) with 30-s chair stand test; ** Not including the 30-s chair stand because of fits level of its correlation (Pearson correlationcoefficient = 0.71) with the Berg Balance Scale; *p* = level of significance.

Totally 88.7% of the participants’ MMSE scores fell within mild and moderate dementia (10–26 points) [70]; 10.9% (n = 16) scored less than 10 points (indicating severe dementia) and 1.4% scored higher than 26 points. 

The score on the QUALID ranged from 11 (n = 12) to 41 (n = 1) points. The mean values for the whole sample, women and men, were 18.1 (SD = 5.8), 18.3 (SD = 5.8) and 17.5 (SD = 5.6), and no significant gender difference was observed regarding QUALID (*p* = 0.44).

Regarding the physical performance tests, the mean values of the tests and standard deviations are shown in Table 1. The results show that the participants differed greatly on the physical performance tests. The mean score on BBS was 34.7 for the whole sample, and the range was 3–56 points. On average, the participants were able to stand up 6 times in 30 s, range 0–14 and mean comfortable walking speed was 0.5 m/s, range 0.1–1.4 m/s. The associations between the different physical performance function variables are shown in Table 2. The highest correlation was found between the “30-s chair stand” and BBS, which had consequences for the further analyses (see statistics).

The unadjusted regression analyses showed a significant relationship between QoL and physical function for the variables measuring muscle strength (30-s chair stands) and balance (BBS, Table 3), and the adjusted analysis showed a significant association between QoL and balance (Table 3). Higher scores on the 30-s chair stands and BBS were associated with a better QoL. The beta coefficient of the BBS is approximately the same size as the beta coefficient of the 30-s chair stands. Furthermore, the univariate regression analysis provided a statistical trend, *p* < 0.10, for the association with the comfortable walking speed score as well as the maximum walking speed max (Table 3).

## 4. Discussion

The data showed that nursing-home residents with dementia differed greatly (large SD) in their QoL as measured by QUALID, as well as in their performance on the different physical function tests and are thus a heterogeneous group. Large differences in physical and mental ability among institutional residents have also been recognised by other authors [71].

Our hypothesis was confirmed: QoL among elderly nursing-home residents with dementia is significantly associated with functional performance. To the best of our knowledge, the present study is the first to report that patients lacking balance and with poor lower extremity strength are likely to have a poorer QoL compared to those with better balance and lower extremity strength. However, because this study has a cross sectional design, we cannot establish causality. In other studies, functional status is reported to be by far the most important factor affecting QoL in old age [72]. It is important to note that not all the measurements of physical function (ADL, walking speed) were significantly associated with QoL. However, we observed a possible statistical trend (*p* < 0.10) in the association between QoL and the comfortable walking speed as well as maximum walking speed (see Table 3).

The participants in our study had lower scores on the QUALID, indicating a better QoL compared to the finding of Falk [22] and Barca and colleagues [21]. We did not include residents with severe degrees of dementia, which could explain this difference. Further, in contrast to other studies [21,73,74], we did not observe impaired function in the activities of daily living as being significantly associated with QUALID. However, it should be noted that Barca and colleagues [21] used Lawton and Brody’s Physical Self-Maintenance Scale (PSMS) for their measurement of ADL, and we used BI. One explanation for this disagreement could be that BI is not as sensitive as the PSMS for measuring basic ADL in nursing home patients with dementia. In line with our results, most studies of persons with dementia report no significant association between the degree of cognitive impairment and the QoL, although this has been disputed [75,76,77,78]. 

Many studies show that age is a determinant for self-perceived QoL [79]. In our study, age did not correlate particularly well with QoL. According to Uotinen and colleagues [80], a perceived age (*i.e*., a person’s age as estimated by his or her physical health and probable life expectancy) is possibly a better indicator of QoL than chronological age.

This study has several limitations. The subjects were enrolled during an intervention trial with a strength training exercise, so they were more likely to be fitter and perhaps more engaged than those who would not have agreed to be part of the intervention. Therefore, the associations observed in this study may not be applicable to the overall population of older people living in nursing homes. All participants were suffering from mild or moderate dementia according to score on CDR, however 11% scored lower than 10 points on MMSE which would indicate severe dementia. This means that 16 participants may be wrongly categorized as sufferers of mild/moderate dementia in this study [70], which could influence results. The cross-sectional design of the study does not allow us to draw conclusions about causality. The research physiotherapists who performed the testing could have administered and interpreted the test differently, although before the study was initiated, testers from the different research sites were trained in the testing procedures to ensure high inter-rater reliability. However, the inclusion criteria, which enabled the enrolment of participants with a broad range of mental and functional capacities, enabled the implementation of the procedures in other settings. A further strength is that the study population seems to represent nursing-home residents with respect to age and gender [21,71,81]. Despite the limitations outlined, our findings provide important information about the association between QoL and physical performance in a population of elderly nursing home residents. These results suggest that interventions aiming to increase balance and muscle strength can increase HR-QoL in older people who live in nursing homes.

## 5. Conclusions

Our study has shown that nursing home residents with dementia in the 18 nursing homes we included are a heterogeneous group in terms of QoL and physical function. The better the participants performed on the physical function tests, the higher they scored on QoL. Preventative strategies should at latest be introduced when a nursing home resident show decline in balance and muscle strengt. However, it would be even better to commence physical exercise even earlier to prevent initial deterioration and increase function. Further studies should investigate possible methods to improve physical functioning and thus the QoL of nursing home residents.
